# Molecular Mechanisms in the Etiopathology of Lichen Sclerosus: A Systematic Review

**DOI:** 10.3390/ijms27135968

**Published:** 2026-07-03

**Authors:** Katarzyna Beutler, Sofiia Khimuk, Anastazja Andrusiewicz, Mateusz Mutwicki, Dariya Pozdnyakova, Danuta Nowicka

**Affiliations:** 1 University Centre of General Dermatology and Oncodermatology, Faculty of Medicine, Wroclaw Medical University, 50-556 Wrocław, Poland; kasia.beutler@gmail.com; 2 Faculty of Medicine, Wroclaw Medical University, 50-367 Wrocław, Poland; sofiia.khimuk@student.umw.edu.pl (S.K.); anastazja.andrusiewicz@student.umw.edu.pl (A.A.); mateusz.mutwicki@student.umw.edu.pl (M.M.); dariya.pozdnyakova@student.umw.edu.pl (D.P.)

**Keywords:** lichen sclerosus, immune dysregulation, T cell activation, NF-κB signaling, TGF-β signaling, microRNA, epigenetic regulation, fibroblast activation, extracellular matrix remodeling, fibrosis

## Abstract

Lichen sclerosus (LS) is a chronic inflammatory skin disorder with an incompletely understood molecular pathogenesis. This systematic review aimed to synthesize current evidence on key molecular mechanisms underlying the disease, with a particular focus on immune dysregulation, epigenetic modifications, and tissue remodeling. A structured literature search identified studies employing transcriptomic, epigenetic, and experimental approaches. The strongest evidence consistently supports a central role of immune activation, particularly T cell-mediated responses involving Th1- and Th17-related pathways, accompanied by increased expression of pro-inflammatory cytokines and activation of the NF-κB signaling pathway. Epigenetic and post-transcriptional mechanisms, including dysregulated microRNAs (notably miR-155-5p) and altered DNA methylation patterns, may sustain immune imbalance and fibroblast activation partly via modulation of the FOXO signaling pathway. In parallel, experimental and multi-omics studies highlight enhanced fibroblast activity and extracellular matrix remodeling, largely associated with the TGF-β signaling pathway, linking inflammation with progressive fibrosis. Emerging data also suggest interactions between immune signaling and metabolic alterations, although these findings remain preliminary. Overall, the available evidence indicates that LS may involve a complex interplay between immune, epigenetic, and fibrotic mechanisms. While several molecular pathways and candidate biomarkers have been identified, their clinical relevance requires further validation in larger, well-designed studies.

## 1. Introduction

Lichen sclerosus (LS) is a chronic and idiopathic skin disorder that typically involves genital skin, marked by a progressive, relapsing nature. Extragenital LS is less common (15–20% of all cases) and can affect areas such as the face, neck, and shoulders. The term lichen sclerosus was first used by Hallopeau in 1887. Over the years, it received multiple names, but the final term “lichen sclerosus” was accepted in 1976 by the International Society of the Study of Vulvovaginal Disease [1,2,3]. While research on this topic remains limited, available evidence indicates that genetic factors predispose individuals to developing LS. Additionally, various other triggers may contribute to LS onset, including coexisting autoimmune conditions, medications, physical injury, hormonal factors, increased body weight, and diabetes mellitus [4]. Several infectious agents, including *Borrelia burgdorferi*, Epstein–Barr virus, and human papillomavirus, have been investigated as potential etiopathogenic factors in LS as well [5,6,7,8]. There is also an observed increased risk of genital cancer in female patients affected with vulvar LS. The increase from the population average is estimated to be 2–5% to 65% of those with a vulvar LS background [9].

LS can occur at any age and in both sexes, but the incidence increases with age, and the male-to-female ratio varies between 1:3 and 1:10. Not surprisingly, significant variations in the incidence and prevalence of the disease have been described by sex and age. LS prevalence varies from approximately 0.1% in the pediatric population up to 3% in women older than 80 [6,10]. Although LS occurs most commonly in postmenopausal women, more than half of cases start developing prior to menopause. Nevertheless, the true prevalence of LS is likely higher than reported due to underdiagnosis [11].

Typically, LS begins with hypochromic changes in the mucosa but it eventually leads to significant alteration of the area in which it develops [12]. The clinical manifestation of LS includes pruritus, atrophy, ivory-white patches, and lesions that can evolve into scarring [13,14,15]. The most characteristic localizations of LS are the interlabial sulci, labia minora and labia majora, and clitoris, whereas the vagina is usually spared. Those scars, when located on the genital surface, can lead to vaginal introitus, phimosis and functional impairment, which may be irreversible [16]. These changes may result in a significant reduction in quality of life (QoL). The clinical picture requires differentiation from lichen planus, lichen simplex chronicus, vitiligo, immunobullous disorders such as mucous membrane pemphigoid, and vulvar intraepithelial neoplasia.

Treatment options are scarce mainly because of an incomplete understanding of the genetic, hormonal, immunological and molecular mechanisms which contribute to LS’s pathogenesis. Currently, treatments involve high doses of highly potent corticosteroids, of which the most commonly used is clobetasol propionate 0.05%. Several randomized trials strongly suggest the superiority of clobetasol over other corticosteroids [17]. Although regenerative treatments, including platelet-rich plasma and adipose-derived mesenchymal stem cells, are gaining increasing attention, their clinical use remains constrained by limited evidence, procedural invasiveness, and potential immunological risks [18,19]. However, in recent years, advances in molecule-targeting treatments may offer more variety, effectiveness and safety in accessible therapy strategies [20,21].

The aim of this study was to review the recent literature regarding potential molecular mechanisms involved in LS. Current research in this area explores several molecular targets, including anti-LMOD1 and anti-MYH11 antibodies and B-cell-related pathways such as nuclear factor kappa B (NF-κB), specifically its subunits NFKB1 (p105/p50) and NFKB2 (p100/p52) [22], as well as transforming growth factor beta (TGF-β1) and the chemokine CXCL10 [23].

## 2. Methods

The search was performed on 27 March 2026 in accordance with the Preferred Reporting Items for Systematic Reviews and Meta-Analyses (PRISMA) statement guidelines [24] using the databases PubMed, Scopus and Web of Science. The completed PRISMA 2020 checklist is provided in Supplementary Materials. The search strategy included the following:

For PubMed: (“lichen sclerosus”[Title/Abstract] OR “lichen sclerosus”[MeSH Terms]) AND (pathogenesis[Title/Abstract] OR mechanism*[Title/Abstract] OR molecular[Title/Abstract] OR immun*[Title/Abstract] OR inflamm*[Title/Abstract] OR autoimmune*[Title/Abstract] OR cytokine*[Title/Abstract] OR “gene expression”[Title/Abstract] OR epigen*[Title/Abstract] OR fibrosis[Title/Abstract] OR “oxidative stress”[Title/Abstract]) AND Humans[MeSH Terms] AND English[lang] AND (“2016/01/01”[Date–Publication]: “2025/12/31”[Date–Publication]) AND (“journal article”[Publication Type] NOT “review”[Publication Type])—301 documents.

For Scopus: TITLE-ABS-KEY (“lichen sclerosus”) AND TITLE-ABS-KEY (pathogenesis OR “molecular mechanism*” OR autoimmune* OR cytokine* OR “gene expression” OR epigenetic* OR fibrosis OR “oxidative stress”) AND TITLE-ABS-KEY (human OR humans OR patients OR clinical) AND (PUBYEAR > 2015 AND PUBYEAR < 2026) AND NOT (DOCTYPE, “re”) AND (LIMIT-TO (LANGUAGE, “English”))—439 documents.

For Web of Science: TS=(“lichen sclerosus”) AND TS=(pathogenesis OR “molecular mechanism*” OR mechanism* OR molecular OR immun* OR inflamm* OR autoimmune* OR cytokine* OR chemokine* OR “gene expression” OR transcriptom* OR proteom* OR epigenetic* OR microRNA OR miRNA OR fibrosis OR “extracellular matrix” OR “oxidative stress” OR “immune response”) AND TS=(human OR humans OR patients OR clinical OR biopsy) AND PY=(2016–2025) NOT DT=(Review)—503 documents.

The identified records were first screened by one reviewer based on titles, abstracts, and full texts, and this assessment was then verified by a second reviewer. Only studies meeting all predefined eligibility criteria, established in accordance with the PI(E)COS framework (“Population”, “Intervention”/”Exposure”, “Comparison”, “Outcomes”, and “Study design”) [25], were included, as outlined in Table 1. The selection procedure is presented in detail in the flow diagram shown in the Section 3.

Risk of bias was assessed using the Joanna Briggs Institute (JBI) Critical Appraisal Checklist for Case–Control Studies. For exploratory multi-omics, ex vivo, and mechanistic studies that did not fully correspond to a standard case–control design, the checklist was applied cautiously to the human comparative component.

**Figure 1:**
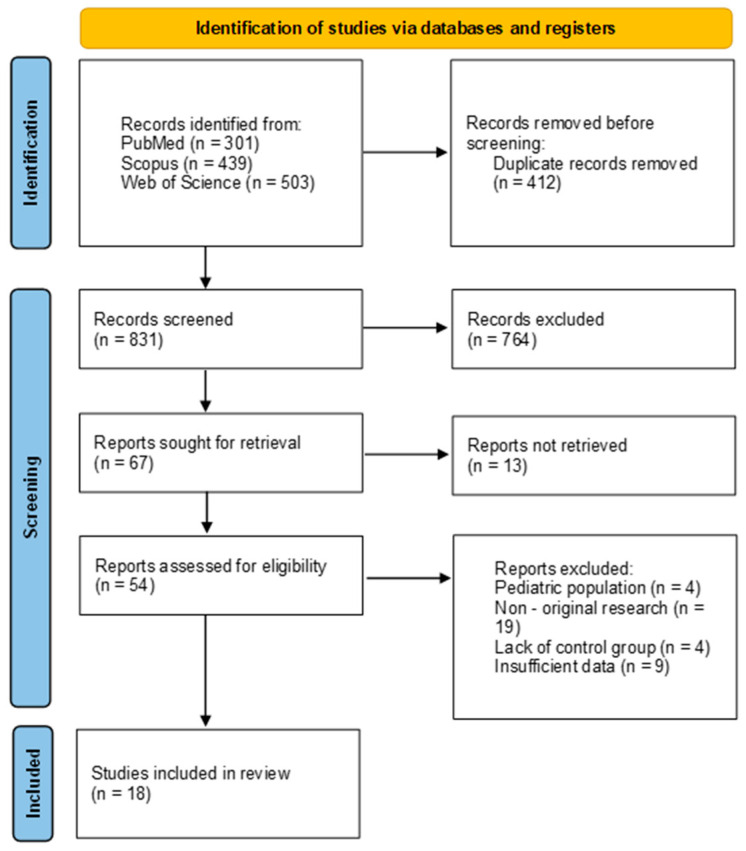
PRISMA flowchart of selected studies.

In the search, we identified 1243 records (301 in PubMed, 439 in Scopus, and 503 in Web of Science). Out of these, 412 duplicate records were removed. A total of 831 titles and abstracts were screened, and out of these 764 were excluded. A total of 67 articles were sought for retrieval, and 54 were assessed for eligibility. Eighteen articles met the inclusion criteria and were summarized in this review. The selection process is presented in the PRISMA flowchart (Figure 1). Full texts could not be obtained despite reasonable retrieval attempts because of the absence of an available online version, incomplete bibliographic information, publication as abstract-only material, unavailable archival content, or unsuccessful attempts to obtain the articles through library services or author contact.

## 3. Results

### 3.1. Characteristics of Included Studies

The eighteen studies included in this review, published between 2018 and 2025, were conducted across multiple geographical regions, including China (seven studies); Poland (four studies); the United States (three studies); and Spain, Italy, and Japan (one study each).

Collectively, the included studies evaluated approximately 291 patients with LS and related conditions and approximately 227 control subjects, although exact numbers varied due to differences in study design, inclusion of disease subgroups (e.g., LS-VSCC and lichen planus), and the absence of control groups in selected studies.

The study designs consisted predominantly of case–control studies (*n* = 9), complemented by mechanistic studies (*n* = 4), multi-omics studies (*n* = 2), one experimental ex vivo study, one proteomic comparative study, and one pilot study. In addition, several studies incorporated in vitro or ex vivo functional experiments, particularly in investigations focused on fibroblast activity, epigenetic regulation, and immune signaling pathways.

Sample sizes varied substantially across the included studies, ranging from very small exploratory cohorts (*n* = 3 patients) to larger case–control studies including up to 49 patients with corresponding control groups. This variability reflects both the rarity of well-characterized LS cohorts and the use of advanced, resource-intensive molecular techniques.

The analyzed biological samples included skin biopsies from vulvar and foreskin tissue, urethral tissue biopsies, peripheral blood, and cultured dermal fibroblasts. These complementary sample types enabled the assessment of both local tissue-specific alterations and systemic immune dysregulation.

Molecular analyses encompassed gene and microRNA expression profiling, DNA methylation studies, proteomics, metabolomics, and integrative multi-omics approaches, targeting pathways associated with immune activation, fibrosis, extracellular matrix remodeling, oxidative stress, and cellular stress responses.

Overall, these heterogeneous methodological approaches provide a comprehensive overview of molecular alterations involved in LS pathogenesis, emphasizing possible interactions between immune dysregulation, epigenetic alterations, and fibroblast-driven tissue remodeling.

The characteristics of the included studies, including participants, molecular focus and principal findings, are summarized in Table 2.

### 3.2. Immune Dysregulation and Inflammatory Pathways

Among the included studies, five focused on immune dysregulation and inflammatory signaling in LS. These studies consistently demonstrated increased expression of pro-inflammatory cytokines, activation of T-cell-mediated responses (including T helper 1 (Th1) and T helper 17 (Th17) pathways), dysregulation of key inflammatory signaling pathways such as NF-κB, and impaired regulatory T-cell function. Collectively, these findings support the involvement of immune imbalance in disease pathogenesis.

In a case–control study, Wierzbicki et al. [22] analyzed foreskin biopsies from 72 adult males, including 49 patients with penile LS (mean age: 50.61 ± 17.65 years), 6 with penile cancer (50.17 ± 11.37 years), 4 with Zoon balanitis (56.5 ± 25.09 years), and 13 controls (28.85 ± 14.39 years). Gene expression analysis was performed using quantitative polymerase chain reaction (PCR) to assess NFKB1 and NFKB2 mRNA levels. The study demonstrated markedly increased NFKB1 expression in penile cancer (approximately 22-fold compared with controls) and in LS, reaching up to approximately 22-fold in advanced stages, with a strong positive correlation with disease severity (rs = 0.667, *p* < 0.0001). NFKB2 expression was also elevated in penile cancer (approximately 3.5-fold compared with controls) but showed no significant association with disease progression. Additionally, micro-incontinence was associated with increased NFKB1 expression in LS (approximately 8-fold). No significant differences in gene expression were observed in Zoon balanitis compared with controls. Overall, these results suggest that activation of the NF-κB pathway, particularly NFKB1, may be involved in inflammation and disease progression in penile LS.

Baran et al. [27] carried out an immunohistochemical analysis using skin biopsies from 20 patients with vulvar LS (20 females, 0 males; mean age: 62.8 years) and 10 healthy controls (10 females, 0 males; mean age: 59.6 years) in a case–control study. The study demonstrated significantly increased expression of interleukin 17 (IL-17) (88.97 ± 20.08 vs. 30.14 ± 14.17 cells; *p* < 0.0001) and S100A7 (67.04 ± 16.16 vs. 14.49 ± 18.31 cells; *p* < 0.0001) in lesional skin compared to controls. Notably, IL-17 was localized in both the epidermis and inflammatory infiltrate, whereas S100A7 was primarily expressed in suprabasal keratinocytes. These observations suggest the possible involvement of Th17-driven inflammation and keratinocyte activation in the pathogenesis of LS.

Czajkowski et al. [29] performed a case–control study using foreskin biopsies from 49 patients with penile LS (49 males, 0 females; mean age: 53.2 years) and 13 healthy controls (13 males, 0 females; mean age: 31.4 years). Gene expression was analyzed using reverse transcription quantitative polymerase chain reaction (RT-qPCR). The study demonstrated markedly increased expression of pro-inflammatory cytokines, including IL-1A (~150-fold), interferon gamma (IFN-γ) (~59-fold), and IL-6 (~7–16-fold), in PLS compared to controls (*p* < 0.05). Cytokine expression varied by disease stage, with IL-1A highest in early PLS and IL-6 highest in severe disease. Micro-incontinence was present in the majority of patients (≈90% vs. 0% in controls; *p* < 0.0001) and was associated with further increases in IL-1A (~400-fold) and IL-6 (~30-fold). Together, these findings suggest that chronic irritation and occlusion may further increase pro-inflammatory cytokine expression in LS.

Wang et al. [37] performed a mechanistic analysis using foreskin tissue from 8 male patients with LS (mean age: 49.4 ± 22.7 years) and 22 normal skin controls from public datasets (age not reported). Integrative DNA methylation and single-cell analysis identified 34,291 differentially methylated probes, predominantly hypermethylated (86.33%; P_adj < 0.05). Cell-type deconvolution revealed a significant reduction in fibroblasts (*p* < 0.001), accompanied by increased T cell infiltration, including CD8+ and regulatory T cells (*p* < 0.05–0.01). Functional enrichment highlighted extracellular matrix organization (P_adj = 8.22 × 10^−5^) and immune activation pathways (P_adj < 0.001), while cell communication analysis identified dominant collagen signaling (*p* < 0.001). Collectively, these findings suggest that epigenetic alterations may be associated with immune–stromal dysregulation in LS.

Wang et al. [39] analyzed vulvar tissue in a mechanistic study, including six patients with vulvar LS (all female; mean age: 55.5 ± 20 years; age of onset: 39.5 ± 17.2 years) and four healthy female controls (mean age: 52.8 ± 23.2 years). RNA sequencing and qRT-PCR validation identified 1641 differentially expressed coding genes (972 increased, 669 decreased; FDR < 0.05, |log2FC| ≥ 1) and 741 non-coding RNAs. Upregulated genes were enriched in T cell activation pathways (e.g., CD3D, CD8B, LCK, and CXCR3/CXCL9–11), while inhibitory NR4A genes were reduced, suggesting an enhanced T cell response. Concurrently, genes related to cell cycle progression (including cyclins and centrosome proteins) were decreased, suggesting impaired proliferation. Pathway analysis identified 16 dysregulated pathways, including activation of Th1/Th2 signaling and inhibition of IL17 signaling. These findings suggest a complex transcriptional imbalance involving immune activation and altered cellular proliferation in vulvar LS.

### 3.3. Epigenetic and Post-Transcriptional Regulation

A subset of five studies addressed epigenetic and post-transcriptional mechanisms, including microRNA expression and DNA methylation. These investigations revealed significant dysregulation of miRNAs and epigenetic modifications affecting genes involved in immune response, fibrosis, and cell proliferation. Together, these findings indicate that such regulatory layers may play a role in modulating disease progression and could contribute to malignant transformation.

Kohli et al. [30] conducted a case–control study involving urethral tissue samples from 22 male patients with LS (median age: 53 years) and 27 male controls (median age: 56 years). miRNA expression profiling using RT-qPCR arrays (752 miRNAs), followed by validation analysis, identified 27 differentially expressed miRNAs (FDR < 0.01), including 9 upregulated and 18 downregulated miRNAs. Among these, 15 demonstrated high diagnostic accuracy (AUC > 0.90), with miR-155-5p showing marked upregulation (~11-fold, *p* < 0.001; AUC = 1.0). Validation confirmed differential expression for 13 out of the 15 miRNAs (FDR < 0.10). Functional analyses indicated enrichment in pathways related to immune response, fibrosis, and angiogenesis, suggesting a possible role for miRNA dysregulation in inflammatory and fibrotic processes in LS.

Ren et al. [33] applied a combined case–control and in vitro design, examining vulvar tissue from 20 LS patients and matched normal samples (sex and age not reported). Using qRT-PCR, luciferase assays, and Western blotting, the study demonstrated significant upregulation of miR-155-5p (*p* < 0.05), alongside reduced expression of forkhead box O3 (FOXO3) and cyclin-dependent kinase inhibitor 1B (CDKN1B) (*p* < 0.05). Functional experiments revealed that miR-155-5p overexpression enhanced fibroblast proliferation and S-phase progression (*p* < 0.01), whereas its inhibition produced the opposite effect. Mechanistically, miR-155-5p directly targeted FOXO3 and CDKN1B, suppressing the FOXO signaling pathway. These results suggest that miR-155-5p may promote fibroblast proliferation through the disruption of FOXO-mediated regulatory mechanisms in LS.

Tan et al. [35] performed a mechanistic study using vulvar tissue from 17 female LS patients and matched adjacent normal tissue (age not reported), complemented by peripheral blood samples from 33 LS patients and 23 healthy female controls. RNA sequencing and qRT-PCR identified 170 differentially expressed miRNAs (119 upregulated, 51 downregulated; FDR-adjusted *p* < 0.05, fold change > 1.5), including immune-associated miR-326 and miR-142-5p, with consistent expression patterns also observed in blood. Functional assays showed that miR-142-5p overexpression reduced fibroblast migration (*p* < 0.05), suggesting a possible role in tissue remodeling. Pathway analysis highlighted 14 networks linked to inflammatory responses and protein kinase B (AKT) signaling, although detailed effect sizes and full demographic data were not provided.

Rotondo et al. [34] analyzed tissue specimens in a case–control study, including 20 patients with LS-associated vulvar squamous cell carcinoma (mean age: 75 ± 3 years), 20 with cancer-associated LS (mean age: 62 ± 11 years), 20 with cancer-free LS, and 20 healthy controls (sex not reported; age not reported for cfVLS and controls). Molecular analysis was performed using RT-qPCR and bisulfite sequencing. The study demonstrated significant downregulation of retinoic acid receptor beta (RARβ) expression in LS-associated vulvar squamous cell carcinoma (LS-VSCC) compared to all other groups (3.4–4.8 fold; *p* ≤ 0.005), accompanied by promoter hypermethylation in 90% of LS-VSCC cases versus 25–55% in LS and control groups. Importantly, higher methylation levels (7–9 cytosine–phosphate–guanine sites [CpGs]) were associated with lymph node metastasis and disease recurrence. Additionally, transcription factor Jun (c-Jun) expression was significantly upregulated (up to 4.3-fold; *p* < 0.001), suggesting activation of oncogenic signaling. These findings suggest that epigenetic silencing of RARβ and associated pathway dysregulation may contribute to malignant transformation in LS.

Wang et al. [39] conducted a case–control study including vulvar tissue and peripheral blood from 15 female LS patients (mean age: 51.0 ± 10.3 years) and 25 controls (10 tissue controls, mean age: 57.5 ± 15.3 years; 15 blood controls, mean age: 45.9 ± 6.7 years). Analyses using bisulfite sequencing, Western blotting, and flow cytometry demonstrated reduced forkhead box P3 (Foxp3) expression and a lower proportion of circulating Tregs in LS patients (6.76 ± 1.47% vs. 8.34 ± 1.72%; *p* < 0.05), along with increased DNA methyltransferase 1 (DNMT1) and DNA methyltransferase 3B (DNMT3b) levels in lesional tissue (*p* < 0.05). Although overall promoter methylation remained unchanged (~100%), specific CpG sites (CpG1, CpG4, CpG9, and CpG10) showed increased methylation (*p* < 0.05). A positive correlation between Foxp3 and DNMT1 expression was also observed (r = 0.675, *p* < 0.05). Collectively, these results point to epigenetic alterations affecting Foxp3 and impaired Treg function as contributors to immune dysregulation in LS.

### 3.4. Fibroblast Activation and Extracellular Matrix Remodeling

Among the included studies, five investigated fibroblast activity and extracellular matrix remodeling in LS. These studies demonstrated increased fibroblast activation, enhanced collagen production, and dysregulation of structural proteins, frequently associated with pathways such as TGF-β signaling. Collectively, these findings support the involvement of fibrosis and tissue remodeling as key components of disease progression.

Fischer et al. [23] investigated paired vulvar biopsy samples from eight patients with vulvar LS (eight females; median age: 63 years), comparing scarred and adjacent unscarred tissue in an experimental setting. Primary fibroblasts were stimulated with TGF-β (5 ng/mL), and molecular analyses were performed using qRT-PCR, Western blotting, and ELISA. The study identified upregulation of SMAD family member 3 (SMAD3) in scarred tissue and significantly increased alpha-smooth muscle actin (αSMA) expression and pro-inflammatory mediators (IL-6, IL-8, and PGE2) in scarred fibroblasts following TGF-β stimulation, while these responses were limited in unscarred cells. These effects were significantly reduced by TGF-β receptor inhibition (*p* ≤ 0.05). Functional analyses suggest the involvement of the SMAD-dependent TGF-β pathway. Overall, these findings support a possible role for enhanced TGF-β signaling in fibroblast activation and inflammatory–fibrotic interactions in VLS.

Lin et al. [31] applied an ex vivo multi-omics approach using foreskin samples from 8 patients with LS (all males, age not reported) and 19 controls (all males, age not reported). Single-cell RNA sequencing and integrative analyses were performed. The study identified significant cellular composition changes, including increased T cells and reduced keratinocytes in LS samples. Fibroblast-mediated signaling was enhanced, with increased fibroblast–T cell interactions (~1.5-fold) and fibroblast–keratinocyte interactions (~1.2-fold), involving the collagen–CD44 (*p* < 0.01) and APP–CD74 (*p* < 0.01) pathways. Collagen-related genes were upregulated in fibroblasts (COL1A1 log2FC ~1.03, COL1A2 ~0.72; *p* < 0.001), while CD74 was strongly increased in keratinocytes (log2FC ~3.13; *p* < 0.001). Multi-omics integration identified 19 candidate genes, with GAS1 significantly downregulated (*p* < 0.005), and its knockdown increased COL1A1/COL6A1 expression and TGFB1 levels (*p* < 0.001). Taken together, these findings indicate that fibroblast-driven signaling and immune–fibrotic interactions may be involved in LS pathogenesis.

Utsunomiya et al. [36] employed an experimental in vitro design with ex vivo validation using dermal fibroblasts and skin samples from 23 female patients with LS (mean age: 69.3 ± 2.4 years) and 3 sex-/site-matched controls (age not reported). Gene expression profiling using cDNA microarray identified 3035 differentially expressed genes following extracellular matrix protein 1 (ECM1) silencing (1471 increased, 1564 decreased; FDR ≤ 0.05, fold change ≥ 1.4). Functional analysis highlighted dysregulation of extracellular matrix organization, cell adhesion, and fibrogenic pathways, with 48 key genes linked to structural remodeling. ECM1 knockdown achieved an ~84.7–88.1% mRNA reduction (*p* = 0.0023) and impaired fibroblast migration and contraction. Ex vivo analysis confirmed altered collagen IV expression in 87.0% of LS samples, laminin-332 in 26.1%, and vascular involvement in 100%. These results suggest that ECM1 deficiency may be associated with extracellular matrix disorganization and fibrotic remodeling in LS.

Zhao et al. [40] analyzed vulvar tissue from 15 patients with vulvar LS (mean age: 55.7 ± 4.3 years; range: 34–65; sex not reported) and 10 controls (age not reported; sex not reported) in a case–control study combined with in vitro experiments. Molecular analyses were performed using immunohistochemistry, Western blotting, a cell viability assay, and gene expression analysis. The study demonstrated significantly increased galectin-7 expression in VLS tissue, including >5-fold upregulation compared to controls (*p* < 0.01). In functional assays, galectin-7 reduced fibroblast viability at higher concentrations (>1 µg/mL), with significant effects observed at 2 µg/mL and 5 µg/mL (*p* < 0.05 and *p* < 0.01, respectively). Additionally, galectin-7 was associated with increased expression of collagen I and collagen III in a dose-dependent manner (*p* = 0.034 and *p* = 0.001, respectively). Collectively, these findings point to a potential role of galectin-7 in epidermal–dermal interactions and extracellular matrix alterations in VLS.

Cong et al. [21] explored gene expression patterns in vulvar tissue from three patients with LS (three females, zero males; age not reported) in a pilot study without an external control group, using adjacent normal tissue as a reference. Molecular analysis was performed using qRT-PCR and Western blotting. The study demonstrated consistent downregulation of genes related to smooth muscle (LMOD1 and MYH11), extracellular matrix (TNXB), adipose tissue (PNPLA3, PLIN5, and ACSL1), and innervation (GLI2) in lesional samples, with statistical significance reported for most targets (FDR < 5%). Protein-level validation confirmed decreased expression of selected smooth muscle markers. While these findings suggest that structural tissue remodeling may be involved in LS pathogenesis, they should be interpreted with caution given the very small sample size (*n* = 3).

### 3.5. Integrated Multi-Omics, Metabolic and Proteomic Alterations

Out of the 18 included studies, three applied advanced molecular approaches, including multi-omics, metabolomics, and proteomics, to provide an integrative view of LS pathogenesis. These studies revealed potential interactions between immune activation, metabolic dysregulation, complement pathways, and stress-related protein responses, suggesting the complex and systemic nature of the disease.

Cong et al. [28] employed a multi-omics approach using paired vulvar tissue samples from 12 patients with LS (sex not reported; mean age: 45 years) and 4 healthy controls (sex not reported; mean age: 36 years). Molecular analyses included RNA sequencing, lipidomics, and metabolomics. The study identified 371 upregulated and 331 downregulated genes in lesional tissue, with enrichment of antiviral and innate immune response pathways. Notably, hepatitis C virus-related poly U/UC sequences were significantly enriched in VLS samples. Metabolomic analysis revealed reduced levels of long-chain and very-long-chain fatty acids, along with alterations in glutathione metabolism, including an increased reduced glutathione/oxidized glutathione (GSH/GSSG) ratio. Overall, these findings suggest that virus-related immune activation may be associated with inflammation-driven metabolic dysregulation in LS.

Anitua et al. [26] utilized a proteomic approach based on plasma rich in growth factors (PRGF) derived from three patients with LS (three females, 0 males; mean age: 41 years, range: 20–61) and three healthy controls (one female, two males; mean age: 51 years, range: 33–69). Proteomic analysis was performed using LC–MS with label-free quantification. The study identified increased expression of immune-related proteins, including linker for activation of T cells (LAT) (FC = 11; *p* = 0.02) and heat shock protein 60 (CH60) (FC = 73; *p* < 0.001), along with alterations in complement components. Thermal treatment reduced complement-related proteins (FC ~0.5; *p* < 0.01). These findings suggest possible involvement of immune and complement pathways in LS; however, interpretation is limited by the small sample size and the indirect experimental model.

Marzec et al. [32] analyzed vulvar biopsy samples in a case–control study including 15 patients with LS and 10 with lichen planus (all females; age range: 24–75 years; mean age not reported), along with 14 healthy controls (all females; mean age not reported). Gene expression analysis was performed using RT-qPCR. The study showed significantly increased heat shock protein family A member 1A (HSPA1A) expression in LS compared to controls (~3.8-fold; 7.44 ± 2.16 vs. 5.52 ± 3.18; *p* < 0.05), while heat shock protein family A member 1B (HSPA1B) was elevated but not statistically significant (9.94 ± 6.88 vs. 6.54 ± 3.41), and tumor protein p53 (TP53) showed no significant differences (9.94 ± 1.27 vs. 9.11 ± 1.14). These results suggest that HSP70-related stress-response pathways may be associated with inflammatory mechanisms associated with LS.

### 3.6. Biological Context and Sample Source-Specific Evidence

The included studies represented distinct biological contexts, and their findings were therefore interpreted according to disease site and experimental model. Studies on vulvar LS mainly implicated local immune activation, Th17/IL-17-related inflammation, epigenetic dysregulation, stress-response pathways, and fibroblast-mediated extracellular matrix remodeling [21,23,27,35,36,39,40]. In penile LS, evidence from foreskin samples emphasized NF-κB activation, increased pro-inflammatory cytokine expression, T-cell infiltration, DNA methylation changes, and collagen-related immune–stromal signaling [22,29,31,37]. Studies including LS-associated vulvar squamous cell carcinoma or cancer-associated vulvar LS were considered separately, as findings such as RARβ promoter hypermethylation and c-Jun upregulation may reflect malignant transformation rather than early LS pathogenesis [34]. Finally, experimental, ex vivo, peripheral blood, PRGF-based, and cultured fibroblast models provided mechanistic support for selected pathways but were not considered directly equivalent to tissue-based clinical evidence because of differences in biological material and model assumptions [23,26,33,35,36,40].

### 3.7. Risk of Bias Assessment

The nine included case–control studies generally had a moderate risk of bias. Most studies used clearly defined disease groups and applied standardized laboratory methods, such as immunohistochemistry, RT-qPCR, Western blotting, bisulfite sequencing, or flow cytometry. Several studies also confirmed diagnoses histopathologically, which strengthened the validity of case identification [27,29,30]. The main methodological limitations were related to small sample sizes, lack of formal matching, and limited control for confounding factors. In several studies, cases and controls differed in age, tissue source, clinical context, or specimen processing. Potential confounders such as menopausal status, disease duration, treatment history, comorbidities, anatomical sampling site, and medication exposure were often insufficiently reported or not adjusted [22,32,39]. Overall, the studies provided useful exploratory evidence on molecular and immunological alterations in LS, but the certainty of conclusions is limited by their observational design, small cohorts, and heterogeneous control groups and the absence of multivariable adjustment. Findings from studies that included additional in vitro experiments should be interpreted as mechanistic support rather than direct clinical evidence [33,40].

Both Cong et al.’s 2021 study [28] and Lin et al.’s study [31] used advanced multi-omics methods and included paired or comparative tissue analyses, which strengthened internal biological interpretation; however, both studies were exploratory and limited by small omics sample sizes, incomplete control for clinical confounding, and lack of independent validation. Therefore, their findings should be interpreted as hypothesis-generating mechanistic evidence, with moderate to high risk of bias for clinical or causal conclusions.

Mechanistic studies [35,36,37,38] provided primarily exploratory molecular or mechanistic evidence, with strengths including standardized omics or laboratory methods, histopathological confirmation in most clinical samples, and selected validation experiments. However, clinical inference was limited by small sample sizes; incomplete matching or use of external controls; limited control for confounding; and, for mechanistic inference, reliance on in vitro or bioinformatic models that cannot fully reproduce lichen sclerosus pathology.

Several other studies [21,23,26] were appraised descriptively only because no JBI checklist fully captures exploratory proteomic ex vivo studies. Table 3 presents a general overview of the risk of bias assessment, while detailed assessment is presented in Appendix A Table A1.

## 4. Discussion

This systematic review provides an integrated overview of molecular mechanisms associated with LS, supporting a multifactorial model in which immune dysregulation, epigenetic alterations, and progressive tissue remodeling may interact during disease development. Despite heterogeneity in study design and scale, several consistent patterns emerge, particularly from studies employing mechanistic and multi-omics approaches, which provide important exploratory insights into disease biology.

Our findings are consistent with the current view that LS is not to be explained by a single molecular defect, but rather by overlapping immune, stromal, epigenetic, and metabolic abnormalities [6,41]. LS is currently described as a chronic inflammatory disease with autoimmune features, Th1-skewed immune activation, cytokine dysregulation, extracellular matrix remodeling, scarring, and risk of malignant transformation; in this context, our review further supports the relevance of T-cell-mediated pathways, NF-κB signaling, IL-17-related inflammation, impaired regulatory T-cell function, and altered cytokine expression [16,42]. These immune findings were accompanied by epigenetic and post-transcriptional changes, including miR-155-5p, Foxp3-related pathways, DNA methylation, and RARβ promoter hypermethylation, suggesting potential links between chronic inflammation, immune persistence, fibrosis, and malignant transformation. Fibroblast dysfunction and extracellular matrix remodeling also emerged as recurring themes, with recurrent involvement of collagen organization, TGF-β/SMAD signaling, ECM1-related alterations, galectin-7, and fibroblast–immune interactions, supporting sclerosis as a potentially active remodeling process. Multi-omics, metabolomic, and proteomic studies further suggested contributions of antiviral and innate immune signatures, lipid and glutathione metabolism, complement changes, and stress-response pathways, although these findings remain exploratory [43,44].

**Figure 2:**
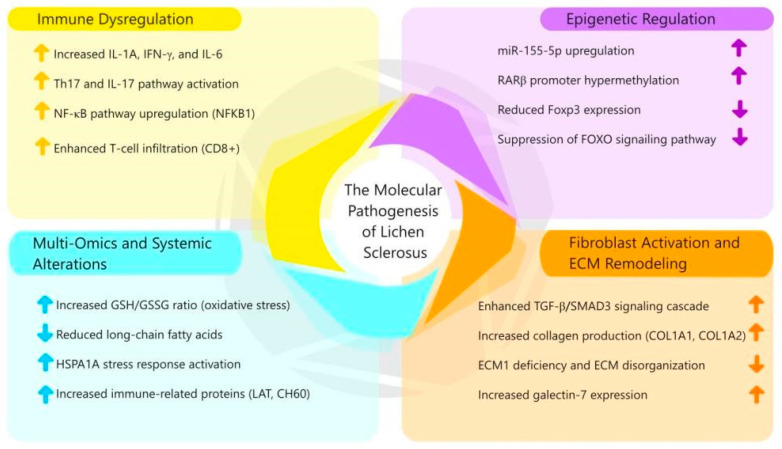
Molecular mechanisms underlying the pathogenesis of lichen sclerosus.

The most consistent molecular pattern that emerges from the included evidence is immune dysregulation. Across studies, LS was associated with increased pro-inflammatory cytokine expression, enrichment of T cell activation pathways, IL-17-related inflammation, and NF-κB signaling. These findings suggest a persistent inflammatory microenvironment with impaired immune regulation, although they should be interpreted mainly as associative molecular signatures rather than definitive causal mechanisms [27,29,37,38]. Epigenetic and post-transcriptional alterations may provide an additional regulatory layer linking immune activation with fibroblast activity and disease persistence. Altered miRNA expression, DNA methylation changes, and pathways related to immune regulation, cell proliferation, and fibrosis were reported, with miR-155-5p- and FOXO-related signaling being notable but still insufficiently validated. Findings from LS-associated vulvar squamous cell carcinoma should be interpreted separately, as they may reflect malignant transformation rather than early LS pathogenesis [30,33,34,35,39]. Fibroblast activation and extracellular matrix remodeling were also recurring themes, involving TGF-β/SMAD signaling, collagen-related pathways, ECM1-associated alterations, galectin-7, and fibroblast–immune interactions. These findings support the view that sclerosis in LS may reflect active stromal remodeling rather than passive scarring alone, but evidence from ex vivo and in vitro models should be regarded as mechanistic support rather than direct clinical evidence [21,23,31,36,40]. Finally, exploratory multi-omics, metabolomic, and proteomic studies suggested links between immune activation, metabolic alterations, complement activity, oxidative balance, and cellular stress responses. However, because these observations are based on small exploratory cohorts or indirect models, they should be considered hypothesis-generating and require validation in standardized, site-specific cohorts [26,28,32]. Taken together, the available evidence suggests that LS may involve interactions between immune activation, epigenetic regulation, and fibroblast-mediated tissue remodeling. Although several pathways and candidate biomarkers have been identified, their clinical applicability remains preliminary and requires validation in larger, well-characterized cohorts. The principal molecular and immunological mechanisms potentially involved in LS are summarized in Figure 2.

This review has several limitations that should be considered when interpreting the findings. First, the available evidence is heterogeneous and includes small case–control studies, pilot studies, in vitro and ex vivo experiments, multi-omics analyses, and proteomic studies. These differences in study design, biological material, analytical methods, and outcome reporting limit direct comparability across studies and preclude quantitative synthesis. Moreover, many findings have not yet been independently validated in larger cohorts and, therefore, should be interpreted as hypothesis-generating rather than definitive evidence of causal mechanisms in lichen sclerosus.

Second, the review process itself had methodological constraints. The number of reviewers involved in the study selection was limited, which may have increased the risk of selection bias. In addition, the inclusion criteria were restricted to English-language publications. This may have introduced language bias and could have led to the omission of relevant studies published in other languages. Another limitation is that 13 potentially relevant reports could not be retrieved despite reasonable attempts; therefore, a degree of retrieval bias cannot be excluded.

Risk of bias assessment was limited by the methodological heterogeneity of the included studies, particularly the presence of exploratory molecular, multi-omics, ex vivo, and in vitro designs for which standard clinical appraisal checklists are not fully applicable. Therefore, although the closest available JBI tools were used where possible, the accuracy and comparability of the risk of bias ratings should be interpreted with caution.

## 5. Conclusions

This systematic review highlights that LS may involve a complex interplay between immune activation, epigenetic dysregulation, and fibroblast-mediated tissue remodeling. The most consistent evidence supports the involvement of T cell-mediated inflammation, associated with activation of the NF-κB signaling pathway and dysregulated cytokine expression. In parallel, epigenetic mechanisms, including altered microRNA profiles and DNA methylation patterns, appear to contribute to persistent immune imbalance and enhanced fibroblast activity, partly through pathways such as the FOXO signaling pathway. Fibrotic remodeling, associated with the TGF-β signaling pathway, further links chronic inflammation with structural tissue changes. Although several molecular pathways and candidate biomarkers have been identified, their clinical applicability remains limited due to the heterogeneity and scale of available studies. Future research integrating multi-omics approaches with well-characterized clinical cohorts is needed to validate these mechanisms and to support the development of targeted diagnostic and therapeutic strategies.

## Figures and Tables

**Table 1:** Eligibility criteria according to PI(E)COS framework.

Parameter	Inclusion Criteria	Exclusion Criteria
Population	Human participants with clinically and/or histopathologically confirmed lichen sclerosus. Adult individuals (≥18 years of age).	Studies including participants under 18 years of age or non-human subjects.
Intervention/Exposure	Studies investigating molecular mechanisms involved in the etiopathology of lichen sclerosus, including but not limited to genetic, epigenetic, immunological, inflammatory, and autoimmune factors	Studies not investigating the molecular mechanisms of lichen sclerosus. Investigations limited to in vitro models without human samples.
Comparison	Healthy controls, unaffected tissue, or comparative groups without lichen sclerosus, where applicable.	Studies without a relevant comparator or control group were excluded when comparison was required.
Outcomes	Identification and characterization of molecular pathways, biomarkers, gene expression profiles, immune responses, and other mechanisms contributing to the development and progression of lichen sclerosus.	Studies not reporting molecular, genetic, or immunological outcomes related to etiopathogenesis. Studies reporting only clinical outcomes without any molecular analysis.
Study design	Original research articles, including observational studies (case–control, cohort, cross-sectional) and experimental studies.	Full version of the document not available. Non-English-language publications. Published before 2016. Literature reviews, editorials, commentaries, letters to the editor, duplicates, and case reports.

**Table 2:** Characteristics of included studies.

Author	Study Design	Study Population	Sample Source	Molecular Target
Anitua et al. [26] Spain 2024	Proteomic study	3 patients with LS, 3 controls	Peripheral blood	LAT, HSP60/CH60, complement proteins; LC–MS
Baran et al. [27] Poland 2024	Case–control	20 patients with VLS, 10 controls	Skin biopsy (vulva)	IL-17, S100A7
Cong et al. [21] China 2020	Pilot study	3 female patients with LS	Skin biopsy (vulva; lesional vs. adjacent tissue)	LMOD1, MYH11
Cong et al. [28] China 2021	Exploratory paired multi-omics study	12 LS patients and 4 controls	Skin biopsy (vulva)	HCV poly U/UC sequences, lipid metabolites, glutathione
Czajkowski et al. [29] Poland 2022	Case–control	49 PLS patients, 13 controls	Skin biopsy (foreskin)	IL-1A, IFN-γ, IL-6
Fischer et al. [23] USA 2025	Experimental study	8 VLS patients	Skin biopsy (vulva; paired scarred and unscarred tissue)	TGF-β, αSMA, IL-6
Kohli et al. [30] USA 2021	Case–control	22 LS patients, 27 controls	Tissue biopsy (urethra)	miRNAs
Lin et al. [31] China 2025	Multi-omics study	8 LS patients, 19 controls	Skin biopsy (foreskin)	GAS1, COL1A1, COL6A1, CD44
Marzec et al. [32] Poland 2023	Case–control	15 LS patients, 10 LP, 14 controls	Skin biopsy (vulva)	HSPA1A, HSPA1B, TP53
Ren et al. [33] China 2018	Case–control	20 VLS patients	Skin biopsy (vulva)	miR-155-5p, FOXO3, CDKN1B
Rotondo et al. [34] Italy 2018	Case–control	20 LS-VSCC, 20 caVLS, 20 cfVLS, 20 controls	Skin biopsy (vulva)	RARβ, c-Jun
Tan et al. [35] USA 2021	Mechanistic study	33 LS patients and 23 controls (blood)	Skin biopsy (vulva) Peripheral blood	miRNAs, IPA pathways
Utsunomiya et al. [36] Japan 2020	Mechanistic study	23 patients with VLS, 3 controls	Skin biopsy (vulva) Dermal fibroblasts (cell culture)	ECM1, laminin-332, collagen-IV
Wang et al. [37] China 2025	Mechanistic study	8 LS patients; 22 healthy controls	Skin biopsy (foreskin)	DNA methylation, T cells, fibroblasts
Wang et al. [38] China 2022	Mechanistic study	6 patients with VLS, 4 healthy controls	Skin biopsy (vulva)	T cell activation genes, CXCL9/ CXCL10, NR4A genes
Wang et al. [39] China 2021	Case–control	15 VLS patients, 25 healthy controls	Skin biopsy (vulva) Peripheral blood	Foxp3, DNMT1, Tregs
Wierzbicki et al. [22] Poland 2022	Case–control	49 PLS and 13 controls	Healthy men	Skin biopsy (foreskin)
Zhao et al. [40] China 2018	Case–control	15 VLS and 10 controls	Patients without LS	Skin biopsy (vulva)

αSMA—alpha-smooth muscle actin; ACSL1—acyl-CoA synthetase long-chain family member 1; AKT—protein kinase B; APP—amyloid beta precursor protein; caVLS—cancer-associated vulvar lichen sclerosus; CD3D—CD3 delta subunit of T-cell receptor complex; CD44—CD44 molecule (cell surface glycoprotein); CD74—major histocompatibility complex class II invariant chain; CD8B—CD8 beta chain; CDKN1B—cyclin dependent kinase inhibitor 1B (p27^Kip1^); CH60—heat shock protein family D member 1 (HSPD1; Hsp60); c-Jun—transcription factor Jun; cfVLS—cancer-free vulvar lichen sclerosus; COL1A1—collagen type I alpha 1 chain; COL1A2—collagen type I alpha 2 chain; COL6A1—collagen type VI alpha 1 chain; CXCL9—C-X-C motif chemokine ligand 9; CXCL10—C-X-C motif chemokine ligand 10; CXCL11—C-X-C motif chemokine ligand 11; CXCR3—C-X-C motif chemokine receptor 3; DNMT1—DNA methyltransferase 1; DNMT3B—DNA methyltransferase 3 beta; ECM1—extracellular matrix protein 1; FOXO3—forkhead box O3; Foxp3—forkhead box P3; GAS1—growth arrest specific 1; HCV—hepatitis C virus; HSP60—heat shock protein 60; HSPA1A—heat shock protein family A member 1A (Hsp70); HSPA1B—heat shock protein family A member 1B (Hsp70); 151 IFN-γ—interferon gamma; IL-1A—interleukin 1 alpha; IL-6—interleukin 6; IL-17—interleukin 17A; IPA—Ingenuity Pathway Analysis; LAT—linker for activation of T cells; LC–MS—liquid chromatography–mass spectrometry; LMOD1—leiomodin 1; LP—lichen planus; LS—lichen sclerosus; LS-VSCC—lichen sclerosus-associated vulvar squamous cell carcinoma; miR-155-5p—microRNA 155-5p; miRNAs—microRNAs; MYH11—myosin heavy chain 11; NFKB1—nuclear factor kappa B subunit 1; NFKB2—nuclear factor kappa B subunit 2; NR4A—nuclear receptor subfamily 4 group A; PLS—penile lichen sclerosus; RARβ—retinoic acid receptor beta; S100A7—S100 calcium-binding protein A7 (psoriasin); TGF-β—transforming growth factor beta; TP53—157 tumor protein p53; Tregs—regulatory T cells; U/UC—uridine/uridine–cytidine; VLS—vulvar lichen sclerosus.

**Table 3:** Risk of bias assessment of included studies using JBI critical appraisal tools.

Author	Study Design	Level of Risk of Bias
Anitua et al. [26]	Proteomic study	NA
Baran et al. [27]	Case–control	Moderate
Cong et al. 2020 [21]	Pilot study	NA
Cong et al. 2021 [28]	Multi-omics study	Moderate to high risk
Czajkowski et al. [29]	Case–control	Moderate
Fischer et al. [23]	Experimental study	NA
Kohli et al. [30]	Case–control	Moderate
Lin et al. [31]	Multi-omics study	Moderate
Marzec et al. [32]	Case–control	Moderate to high risk
Ren et al. [33]	Case–control	Moderate
Rotondo et al. [34]	Case–control	Moderate to high risk
Tan et al. [35]	Mechanistic study	Moderate
Utsunomiya et al. [36]	Mechanistic study	Moderate
Wang et al., 2025 [37]	Mechanistic study	Moderate to high risk
Wang et al., 2022 [38]	Mechanistic study	Moderate
Wang et al., 2021 [39]	Case–control	Moderate
Wierzbicki et al. [22]	Case–control	Moderate
Zhao et al. [40]	Case–control	Moderate

## Data Availability

Data supporting the findings of this study are available within the article.

## Supplementary Materials

The following supporting information can be downloaded at: https://www.mdpi.com/article/10.3390/ijms27135968/s1.

## Abbreviations

The following abbreviations are used in this manuscript:

ACSL1	Acyl-CoA synthetase long chain family member 1
AKT	Protein kinase B
APP	Amyloid beta precursor protein
AUC	Area under the curve
BMI	Body Mass Index
CD3D	CD3 delta chain
CD44	CD44 molecule
CD74	CD74 molecule
CD8B	CD8 beta chain
CD8+	Cluster of differentiation 8 positive
CH60	Heat shock protein 60
COL1A1/1A2/6A1	Collagen type I alpha 1/alpha 2/type VI alpha 1
CpG	Cytosine–phosphate–guanine
DNA	Deoxyribonucleic acid
DNMT1/3B	DNA methyltransferase 1/3B
ECM1	Extracellular matrix protein 1
FC	Fold change
FDR	False discovery rate
FOXO/FOXO3	Forkhead box O/forkhead box O3
FoxpOXP3	Forkhead box P3
GAS1	Growth arrest specific 1
GLI2	GLI family zinc finger 2
GSH/GSSG	Reduced glutathione/oxidized glutathione
HSPA1A/1B	Heat shock protein family A member 1A/1B
IFN-γ	Interferon gamma
IL-1A/6/8/17	Interleukin 1 alpha/6/8/17
LAT	Linker for activation of T cells
LC–MS	Liquid chromatography–mass spectrometry
LCK	Lymphocyte-specific protein tyrosine kinase
LMOD1	Leiomodin 1
LS	Lichen sclerosus
miRNA/miRNAs	microRNA/microRNAs
miR-155-5p/142-5p/326	microRNA 155-5p/142-5p/326
MYH11	Myosin heavy chain 11
NF-κB	Nuclear factor kappa B
NFKB1/2	Nuclear factor kappa B subunit 1/2
NR4A	Nuclear receptor subfamily 4 group A
PGE2	Prostaglandin E2
PLIN5	Perilipin 5
PNPLA3	Patatin-like phospholipase domain containing 3
PRGF	Plasma rich in growth factors
PCR/qPCR/RT-qPCR	Polymerase chain reaction/quantitative polymerase chain reaction/reverse Transcription quantitative polymerase chain reaction
QoL	Quality of life
RARβ	Retinoic acid receptor beta
RNA	Ribonucleic acid
rs	Spearman’s correlation coefficient
SMAD3	SMAD family member 3
TGF-β/TGF-β1	Transforming growth factor beta/transforming growth factor beta 1
TGFB1	Transforming growth factor beta 1
Th1/2/17	T helper 1/2/17
TNXB	Tenascin XB
TP53	Tumor protein p53
Treg/Tregs	Regulatory T cell/regulatory T cells
αSMA	Alpha smooth muscle actin

## Appendix A

**Table A1:** Risk of bias assessment tables for included studies.

Study	Q1	Q2	Q3	Q4	Q5	Q6	Q7	Q8	Q9	Q10
**Risk of Bias Assessment Using the JBI Case–Control Checklist**										
Baran	Unclear	Yes	No	Yes	Yes	Partly Yes	Partly Yes	Yes	NA	Yes
Czajkowski	Partly Yes	No	Yes	Yes	Yes	Partly Yes	Partly Yes	Yes	NA	Yes
Kohli	Partly Yes	No	Yes	Yes	Yes	Partly Yes	Partly Yes	Yes	NA	Yes
Marzec	Unclear	No	Partly Yes	Yes	Yes	Partly Yes	Partly Yes	Yes	NA	Partly Yes
Ren	Yes	Yes	Unclear	Yes	Yes	No	No	Yes	NA	Partly Yes
Rotondo	Partly Yes	No	Partly Yes	Yes	Partly Yes	Partly Yes	No	Yes	NA	Partly Yes
Wang 2021 [39]	Partly Yes	No	Partly Yes	Yes	Partly Yes	Partly Yes	Partly Yes	Yes	NA	Partly Yes
Wierzbicki	Partly Yes	No	Yes	Yes	Yes	Partly Yes	Partly Yes	Yes	NA	Partly Yes
Zhao	Partly Yes	No	Yes	Yes	Yes	Partly Yes	No	Yes	NA	Partly Yes
**Risk of Bias Assessment of Multi-Omics Studies Using the JBI Case–Control Checklist**										
Cong 2021	Partly Yes	Yes	Partly Yes	Yes	Yes	Partly Yes	Partly Yes	Yes	NA	Partly Yes
Lin	Partly Yes	No	Partly Yes	Yes	Partly Yes	Partly Yes	Partly Yes	Yes	NA	Partly Yes
**Risk of Bias Assessment of Mechanistic Studies Using the JBI Case–Control Checklist**										
Tan	Partly Yes	Yes	Partly Yes	Yes	Yes	Partly Yes	Partly Yes	Yes	NA	Partly Yes
Utsunomiya	Partly Yes	Partly Yes	Partly Yes	Yes	NA	Partly Yes	Partly Yes	Yes	NA	Partly Yes
Wang 2025 [37]	No	No	Partly Yes	Yes	Partly Yes	Partly Yes	Partly Yes	Yes	NA	Partly Yes
Wang 2022 [38]	Partly Yes	Partly Yes	Partly Yes	Yes	Yes	Partly Yes	Partly Yes	Yes	NA	Partly Yes

Checklist questions:
Q1. Were the groups comparable other than the presence of disease in cases or the absence of disease in controls?
Q2. Were cases and controls matched appropriately?
Q3. Were the same criteria used for identification of cases and controls?
Q4. Was exposure measured in a standard, valid and reliable way?
Q5. Was exposure measured in the same way for cases and controls?
Q6. Were confounding factors identified?
Q7. Were strategies to deal with confounding factors stated?
Q8. Were outcomes assessed in a standard, valid and reliable way for cases and controls?
Q9. Was the exposure period of interest long enough to be meaningful?
Q10. Was appropriate statistical analysis used?
